# Regression Analysis of Perceived Stress among Elite Athletes from Changes in Diet, Routine and Well-Being: Effects of the COVID-19 Lockdown and “Bubble” Training Camps

**DOI:** 10.3390/ijerph19010402

**Published:** 2021-12-30

**Authors:** Jad Adrian Washif, Achraf Ammar, Khaled Trabelsi, Karim Chamari, Christabelle Sheau Miin Chong, Siti Fuzyma Ayu Mohd Kassim, Philip Chun Foong Lew, Abdulaziz Farooq, David B. Pyne, Carl James

**Affiliations:** 1 Sports Performance Division, Institut Sukan Negara Malaysia (National Sports Institute of Malaysia), National Sports Complex, Kuala Lumpur 57000, Malaysia; christabelle@isn.gov.my (C.S.M.C.); fuzyma@isn.gov.my (S.F.A.M.K.); chun_foong@isn.gov.my (P.C.F.L.); carl@isn.gov.my (C.J.); 2 Department of Training and Movement Science, Institute of Sport Science, Johannes Gutenberg-University Mainz, 55099 Mainz, Germany; ammar1.achraf@ovgu.de; 3 Institute of Sport Science, Otto-von-Guericke University, 39106 Magdeburg, Germany; 4 Interdisciplinary Laboratory in Neurosciences, Physiology and Psychology: Physical Activity, Health and Learning (LINP2), UPL, Paris Nanterre University, 92000 Nanterre, France; 5 High Institute of Sport and Physical Education, University of Sfax, Sfax 3000, Tunisia; trabelsikhaled@gmail.com; 6 Research Laboratory: Education, Motricity, Sport and Health, EM2S, LR19JS01, University of Sfax, Sfax 3000, Tunisia; 7 Aspetar, Orthopaedic and Sports Medicine Hospital, FIFA Medical Centre of Excellence, Doha 29222, Qatar; karim.chamari@aspetar.com (K.C.); mohammed.farooq@aspetar.com (A.F.); 8 Research Institute for Sport and Exercise, University of Canberra, Canberra 2617, Australia; David.Pyne@canberra.edu.au

**Keywords:** detraining, emotion, mental health, Olympic, Paralympic, perception, quarantine, remote coaching, sports nutrition, training camp

## Abstract

The COVID-19 pandemic has affected the lifestyles and training of elite athletes around the world. The detrimental effects of lockdown periods may vary among individuals, as well as among sports and sexes. This study investigated the changes in dietary habits, and the predictors of perceived stress during lockdown and a “bubble” training camp. This cross-sectional, online survey involved 76 elite and world-class athletes from six able-bodied sports and nine parasports, all of whom were involved in a 30-day “bubble” training camp. Questions were asked on socio-demographics, training routines and wellbeing, perceived stress, and dietary habits, pertaining to “normal” training (prelockdown), lockdown training, and “bubble” camp training periods. Changes in perceived stress were *trivial* to *small* during lockdown compared to “normal” training, and *trivial* to *moderate* during a “bubble” camp, compared to lockdown. Para-athletes, males, older athletes, less experienced athletes, married individuals, and specific ethnicities appeared to be more detrimentally affected (increased perceived stress) by lockdown. These negative experiences, however, were largely reversed during “bubble” camps. During lockdown, more athletes reported increased evening snack consumption (+8%), later meal-times (+6%), decreased fluid intake (−6%), and no breakfast (+7%). These changes were reversed during “bubble” camps (12–18% improvements). Sport classification accounted for 16% of the increased perceived stress (*p* = 0.001) during lockdown. Overall, socio-demographic factors, improvements in training routines, well-being, and dietary habits explained 28% of the decreased perceived stress during a “bubble” camp. In conclusion, better dietary habits, training routines and well-being have implications for reduced perceived stress. During lockdown, “bubble” camps may be beneficial, but this observation may be a case-by-case consideration, and short split “bubble” periods are recommended.

## 1. Introduction

The coronavirus disease 2019 (COVID-19) pandemic has affected the ability of many athletes to travel, train and compete. There is emerging evidence concerning the detrimental physical and mental effects of the pandemic [1,2], including on athletic populations [3,4]. The detrimental effects of lockdowns appear to vary among individuals [5]. For example, para- and able-bodied athletes require differing levels of support and equipment to train effectively [6].

During lockdown, an increased incidence of psychological issues has been reported. For example, depression and anxiety have increased significantly in many countries [1,2,3,4,6,7]; the effects of these issues must not be underestimated [8]. Unlike mental health, other clinical issues, such as musculoskeletal injury, were not severely aggravated by lockdowns [9]. Psychological effects appear to be influenced by sex, with females, in general, demonstrating greater COVID-19-related fear [8] and stress [10]. Female athletes also reported greater perceived stress than males during lockdown [11,12]. Lockdown-mediated psychological impacts have the potential to be wide-ranging, substantial, and long lasting [13]. Thus, increased understanding of the factors that may act as stressors is warranted in order to support the wellbeing and training needs of athletes. While many consequences of the pandemic have affected both the general and athletic populations [2,3,14,15], limited research has specifically investigated stressors among elite athletes.

Lifestyle changes during COVID-19 lockdowns have influenced both sleeping and dietary patterns, through circadian misalignment, causing alterations in eating and sleeping behaviours [16,17]. During lockdown, movement restrictions have limited food choices, led to unfavourable food choices and increased food consumption [18]. Increased consumption of late-night snacks and “skipping breakfast” appear to be further dietary consequences of lockdowns [19]. In Rugby, researchers underlined the importance of appropriate food intake to maintain performance during lockdown periods [20]. Moreover, within the wider population, research has shown increased stress levels among adult females to be associated with poor eating behaviours [21]. However, limited studies have considered whether this relationship also applies to elite athlete populations during COVID-19 lockdowns.

In order to mitigate the effects of lockdowns on athletes’ training routines, wellbeing and nutrition, “bubble” training camps have emerged in many countries. A “bubble” camp can be defined as a quarantine-style camp whereby a group of people (e.g., athletes, support staff) is strategically isolated from wider society to maintain/resume regular activities for a specified period of time [22]. In Malaysia, specific “bubble” training camps during the early COVID-19 pandemic have enabled elite athletes to resume “normal” training practices, utilising specific training facilities and support staff, as well as healthy food choices. These arrangements had positive effects on athletes’ routines, wellbeing, sleep behaviours, and perceived stress [22]. Favourable outcomes were likely due to a combination of the reinstatement of a typical training week, support staff availability, as well as appropriate recovery modalities and food intake. Despite the favourable effects of “bubble” training camps on elite athletes, it is unclear which factors were most beneficial for the resulting improvements in athlete stress levels and dietary practices.

Therefore, we investigated (a) changes in dietary habits, and (b) predictors of changes in perceived stress during a COVID-19 lockdown and subsequent “bubble” training camps. We hypothesised that dietary habits would be negatively affected during lockdown, but improved during a “bubble” camp. Further, we hypothesised that socio-demographic factors, negative training routines and wellbeing, and dietary habits would contribute to increased stress levels among elite athletes.

## 2. Materials and Methods

Data resulting from a cross-sectional, online survey study was used to analyse the predictive factors contributing to changes in perceived stress levels and dietary habits across prelockdown, lockdown, and “bubble” camp training phases. All data were recorded within a 5-day period following the completion of the 30-day “bubble” camps (throughout June 2020) in Malaysia, with the same questions being asked for each of the three periods within the framework of the survey. Data were collected using a customised Google Form. In this study, we analysed questions on dietary habits, and utilised previously published data [22] to inform a wide-range of predictive factors related to changes in perceived stress levels. To control for potentially untruthful responses from athletes, we engaged with coaches, trainers, and scientists to assist in guiding all participating athletes to provide considered, truthful responses in order to obtain trustworthy data.

### 2.1. Participants

**Figure 1:**
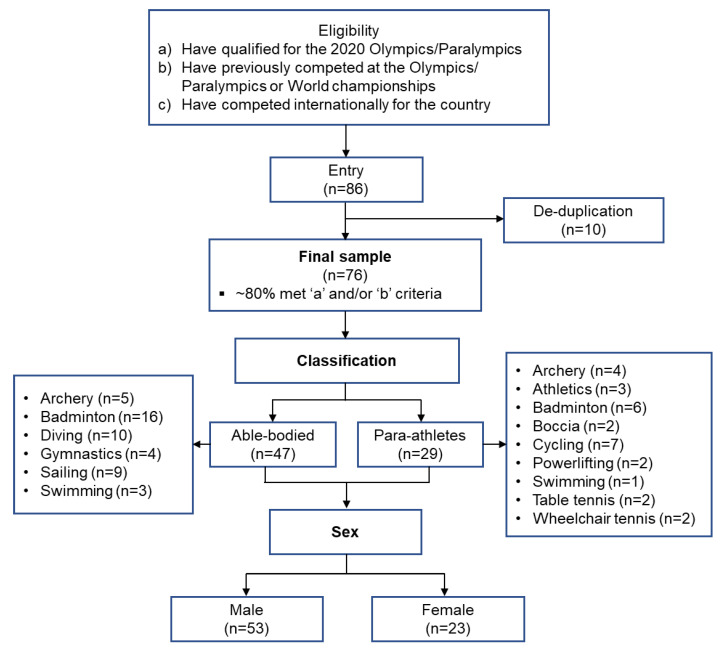
Flow chart of the study’s sample recruitment.

Seventy-six elite Malaysian athletes (26 ± 5 years, range 17–46 years) completed the survey, representing a response rate of 95%. All participants had between 8 and 22 years of competitive experience in their sports (15.5 ± 6.5 years). The inclusion criterion for the study was being a “most elite” level athlete (Figure 1). Participants with intellectual disabilities and athletes who did not participate in the camp were not recruited/allowed to participate. Given the predetermined number of athletes involved in the camps, all athletes were invited to participate in the survey, and thus, no sample size estimation was deemed necessary. Informed consent was obtained from all athletes, with data being processed anonymously. The questionnaires were comparable to those the athletes would routinely provide as part of their official responsibilities as national athletes [23]. The study was conducted according to the Declaration of Helsinki, and received approval by the research committee of Institut Sukan Negara (004/2020-005/2020).

### 2.2. Survey Questionnaire

Participants entered information relating to background and training history, training routines and wellbeing, perceived stress, and dietary habits. Perceived stress was assessed using perceived stress scale (PSS-10), scored from 0 to 40. A higher score indicates a higher perceived stress level, i.e., low stress 0–13, moderate stress 14–26, and high stress 27–40 [24,25]. A more detailed description of the questionnaires is provided in [22]. Dietary habits were assessed through six questions relating to (a) daily amount of water consumption or intake (drink; in litres), (b) frequency of daily meals eaten, (c) weekly frequency of evening meal less than two hours before bed or late dinner, (d) weekly frequency of late-night or evening snacks, and (e) weekly frequency of not consuming breakfast or skip breakfast. Finally, (f) perceived anthropometry (i.e., body mass, which is influenced by dietary intake, included as part of the variables associated with dietary habits) was recorded. Approximately 15 to 20 min was required to complete the full online survey. These questions corresponded to the prelockdown, lockdown, and “bubble” camp training periods. The survey was administered in English and Malay.

### 2.3. Statistical Analysis

Raw data were downloaded from Google Forms and extracted into a Microsoft Excel spreadsheet (Microsoft Corporation, Redmond, WA, USA) for duplication checking and identification of missing data. Data were normally distributed, as confirmed by visual methods (i.e., histograms and Q-Q plots) and/or a Shapiro-Wilks test; therefore, parametric analysis was employed. ANOVA was used to identify differences where there were more than two comparisons (e.g., Malay vs. Chinese vs. Bumiputera vs. Indian). A *t*-test was used to compare means of two comparisons (e.g., able-bodied vs. para-athletes). Cohen’s *d* effect sizes were calculated, and interpreted with the following criteria: <0.2 (as *trivial*), 0.2 (as *small*), 0.5 (as *moderate*) and 0.8 (as *large* change) [26]. Data are presented as mean ± SD.

Multivariate analyses (multiple regressions using sequential modelling, and stepwise backward elimination for an additional/last model) were used to estimate the individual contribution of predictive variables and determine the predictors of changes (delta scores, ∆) in perceived stress (dependent variable) from predictor variables (socio-demographic, training routines and wellbeing, and dietary habits). These analyses were also used to assess the change in R-square (denoted as delta R^2^ or ∆R^2^) in order to identify the degree to which additional variables accounted for the variance in perceived stress [9]. Analyses were conducted for (a) lockdown minus prelockdown, and (b) ‘bubble’ camp minus lockdown. Collinearity of independent variables was verified based on a variance inflation factor threshold of 10 [27,28]. A statistical analysis was performed using IBM SPSS Statistics for Windows, version 26.0 (IBM Corp., Armonk, NY, USA), with the significance level set at *p* ≤ 0.05.

## 3. Results

### 3.1. Changes in Perceived Stress

Responses to the PSS questionnaire are presented in Table 1. Prelockdown data showed a difference between single and married athletes, and lockdown data revealed differences between able-bodied and parasports, and between <10 years and ≥10 years of experience (Table 1). An analysis of subcomparisons showed that PSS changed across training phases, mostly with *small* changes from prelockdown to lockdown, which were reversed (again with *small* changes) during “bubble” camps. Differences in perceived stress were identified between ethnicities. There were increased PSS scores from lockdown to “bubble” camp for East Malaysian Bumiputera, while the PSS scores from Malay, Chinese and Indian decreased (Table 1).

### 3.2. Dietary Habits

The frequency of late-night or evening snack (prelockdown: 2.8 ± 1.0 N·wk^−1^; lockdown: 3.1 ± 1.1 N·wk^−1^; “bubble” camp: 2.5 ± 1.0 N·wk^−1^) eating changed from prelockdown to lockdown (8%, *d* = 0.23, *p* = 0.002), prelockdown to “bubble” camp (−11%, *d* = 0.33, *p* = 0.003), and lockdown to “bubble” camp (−18%, *d* = 0.53, *p* = 0.001). For daily fluid intake (2.2 ± 0.6 L; 2.1 ± 0.8 L; 2.4 ± 0.6 L), changes were evident between prelockdown and lockdown (−6%, *d* = 0.17, *p* = 0.050), prelockdown and “bubble” camp (7%, *d* = 0.25, *p* = 0.008), and between lockdown and “bubble” camp (13%, *d* = 0.40, *p* = 0.001) (Figure 1).

**Figure 2:**
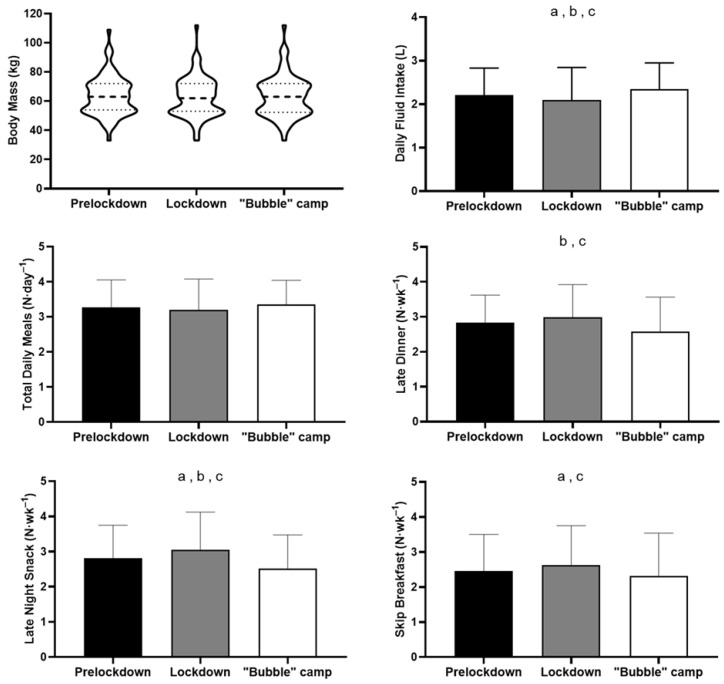
Dietary habits with statistical changes for prelockdown to lockdown (a), prelockdown to “bubble” camp (b), and lockdown to “bubble” camp (c). Note; violin plots show the underlying density distributions of the data.

The frequency of late dinner (2.8 ± 0.8 N·wk^−1^; 3.0 ± 0.9 N·wk^−1^; 2.6 ± 1.0 N·wk^−1^) changed from prelockdown to “bubble” camp (−9%, *d* = 0.28, *p* = 0.010), and lockdown to “bubble” camp (−14%, *d* = 0.43, *p* = 0.001). Similarly, the frequency of skipping breakfast (2.5 ± 1.0 N·wk^−1^; 2.6 ± 1.1 N·wk^−1^; 2.3 ± 1.2 N·wk^−1^) changed for prelockdown to lockdown (7%, *d* = 0.16, *p* = 0.023,), and lockdown to “bubble” camp (−12%, *d* = 0.26, *p* = 0.006). No substantial changes were observed for body mass (*p* = 0.360; 63.5 ± 13.5 kg, 62.8 ± 13.5 kg, 63.2 ± 14.0 kg), for combined male and female athletes, and total daily meals (*p* = 0.400; 3.3 ± 0.8 N·day^−1^; 3.2 ± 0.9 N·day^−1^, 3.4 ± 0.7 N·day^−1^) (Figure 2).

### 3.3. Predictors of Perceived Stress

*Pre-to-during lockdown* (Table 2). In the first, second, and third models, only sport classification was a significant predictor of (increased) perceived stress. Collectively, socio-demographic factors, (negative) training routines, and (negative) wellbeing only explained 27% of the increased perceived stress (i.e., not significant). The addition of eating habits only explained a further 3% of the variance. Additionally, the underlying sport classification contributed to 16% of the change in perceived stress experienced by athletes from pre-to-during lockdown (*p* = 0.001).

*Lockdown-to-“bubble” camp* (Table 3). None of the independent variables was significant in the first, second, and third models. Collectively, socio-demographic factors explained 9% of the decrease in perceived stress (not significant). Adding (positive) training routines and well-being increased the amount of variance explained by the model by 16% (i.e., not significant). The inclusion of dietary habits only increased 3% of the variance. An analysis of stepwise backward elimination indicated that ∆ mental health was a significant contributor to the fourth model. Collectively, the fourth model (including sport classification and ∆ access to sport-specific facilities) accounted for 17% of the change (*p* = 0.004) in perceived stress experienced by athletes from during lockdown-to-“bubble” camp.

## 4. Discussion

This study investigated changes in dietary habits and factors contributing to changes in perceived stress in elite athletes, across different training situations during the COVID-19 pandemic. We found perceived stress among athletes to be *moderate* across prelockdown, lockdown and “bubble” camp training phases. Socio-demographic factors were associated with *trivial* to *small* increases in PSS scores from prelockdown to lockdown, and *trivial* to *large* decreases from lockdown to the “bubble” camp. Lockdown appeared more disadvantageous to para-athletes, males, older athletes, less experienced athletes, married individuals, and specific ethnicities. However, these negative experiences were restored to prelockdown levels during a “bubble” camp. Positive changes in athletes’ dietary habits were also observed during a “bubble” camp.

Increased perceived stress during lockdown was primarily mediated by sport classification, with para-athletes experiencing a *small* deterioration, while stress levels among able-bodied athletes were unchanged. Sport classification explained 16% of the variance for increased PSS scores from prelockdown to lockdown. On the other hand, decreased PSS scores (i.e., improvement) during the “bubble” camp were primarily influenced by several variables (fourth model), i.e., ∆ mental health, sport classification, and ∆ access to sport-specific facilities, which accounted for 17% of the decreased perceived stress. Overall, socio-demographic factors, and improvements in training routines, well-being and dietary habits explained 28% of decreased perceived stress. It is conceivable specific socio-demographic, well-being, and dietary habits influenced the factors that mediated increased or decreased stress levels.

### 4.1. Changes in Perceived Stress

During lockdown, *small* increases in perceived stress were observed among para-athletes, males, athletes aged ≥23 years old, and less-experienced athletes (≤10 years) (Table 1). The changes reported by para-athletes may be partially explained by individuals routinely requiring additional training support. However, such provision was not available or feasible during lockdowns, resulting in modified individual training without the usual care and supervision [6]. Surprisingly, male athletes had a larger increase in perceived stress than female athletes during lockdown, although both reported “moderate stress levels.” In the general adult population, females appear to report greater levels of anxiety and fear than males [29]. Among female athletes, facing circumstances of uncertainty (e.g., about career/future) and economic instability [30] has been associated with increased stress levels. During lockdown, difficulties accessing regular healthcare [31] could potentially elicit stress among female athletes as well. These discrepancies may have been related to the large majority of male athletes (i.e., 90%) within parasports, a group that appeared to have been affected significantly by lockdown (Table 1). Finally, less experienced athletes reported greater stress than more experienced athletes, who might have developed coping mechanisms to regulate stress. For more experienced athletes, a return to “normal” training within a “bubble” was likely a positive opportunity to improve their performance. This opportunity may have helped to minimise uncertainties associated with training and competition, as well as potential loss of income [32].

Despite the apparent support of a family-network and not being isolated, married individuals were more severely affected (*moderate* change) than single individuals during lockdown. Additionally, differences in PSS were identified based on ethnicity during the “bubble” camps (Table 1), possibly reflecting a limitation of a uniform approach for all athletes within the camp. Nevertheless, observations were drawn from uneven sample sizes for ethnicity comparisons. As shown in different countries [1,15], perceived stress was evident during the early stages of lockdown in Malaysia; this worsened as the pandemic continued, i.e., after two months of lockdown [33]. These negative consequences were reduced in most of the athletes once they resumed “normal” training routines during the “bubble” camp, which was held ten weeks after the start of the country’s lockdown.

During lockdown, importantly, perceived stress amongst athletes was typically at a *moderate* level (i.e., indicated by a score within 14–26), with inconsistent baseline levels (varied), apparently influenced by socio-demographic variables. In Italy, prelockdown perceived stress levels of 14 ± 7 in females and 12 ± 6 in males increased to *moderate* levels of 20 ± 6 (female) and 17 ± 6 (male) during lockdown [12]. Such changes are consistent with the data reported in the current study (Table 1). Elite/expert athletes reported lower perceived stress than novice athletes, although all were within the *moderate* range [12]. Collectively, these findings highlight the need for individualised psychological strategies for athletes facing similar challenges in future.

### 4.2. Changes in Dietary Habits

Lockdown had a detrimental effect on dietary habits, with an increased frequency of late-night snacks, late dinners, reduced fluid intake and less breakfast consumption (Figure 2). However, these negative changes were reversed during “bubble” camps. As well as the prevalence of a large variety of healthy food choices, another contributing factor to this improvement may have been the fixed meal schedules in the camps. Neither positive nor negative changes in dietary habits were accompanied by changes in body mass perception (loss or gain). We postulate that athletes have grasped the notion that maintaining an ideal body weight is necessary as an elite athlete, especially when in preparation for the Olympic or Paralympic Games. A global study among general populations showed a widespread adoption of unhealthy eating patterns, relative to prelockdown [18]. Among rugby players, food intake was maintained or increased [20], while among cyclists, food intake appeared to reduce [34]. The emergence of digitally mediated nutritional support should help athletes minimise the negative effects of lockdown related to eating habits via online education/interaction [22,35].

### 4.3. Stress Predictors of Changes between Training Situations

Sport classification appeared to be the single best predictor of increased perceived stress during lockdown (Table 2). Para-athletes displayed a *negative* change in perceived stress, while able-bodied athletes appeared to be unaffected (11% vs. 0%). During lockdown, para-athletes had higher “conscientiousness” than able-bodied athletes, which indicates that they wished to control every aspect of training, nutrition, life, etc. [6]. Coaching elite para-athletes requires a more adaptive approach (e.g., considering the athlete’s unique abilities) [36] that may be challenged by remote working. The current data revealed that 16% of the increase in perceived stress during lockdown was associated with sport classification (Table 2). These findings may also be related to reduced training motivation during lockdown [3,4,22]. Decreased training motivation may impair an individual’s ability to perform the training exercises appropriately and reduce the choice of exercises/activities [37,38].

During lockdown, athletes in different sports may experience a variety of well-being (e.g., mental health) challenges (e.g., [3,22]), although the extent to which these affect their subsequent sporting outcomes (competitions) was not well understood. During the “bubble” camp, the reduced negative well-being (or enhanced mental health) might be interpreted as a positive contributor to improved perceived stress levels (Table 3). Importantly, during the “bubble” camps, access to “normal routines” provided athletes not only with an opportunity to resume their regular activities, such as sport-specific training, but also enabled “normal” interactions with teammates, coaching staff, and performance supports [22], which had been previously limited during lockdown [14]. This positive outcome may be linked with social facilitation, which could substantially benefit para-athletes who were most adversely affected by lockdowns [6]. These findings demonstrate a need for interventions (e.g., “bubble camp” approach) and/or performance support to focus upon supporting athletes (e.g., psychological, nutritional, training perspectives), irrespective of sport classification, during the pandemic when training is interrupted.

### 4.4. Limitations and Strengths of the Study

Data were collected via an online survey, which has the potential to be influenced by untruthful responses. To control for this limitation, we engaged with coaches, trainers, and scientists to assist in guiding all participating athletes to provide considered, truthful responses so that trustworthy data could be derived for the future benefit of all athletes. Our sample size was relatively “small”, as we did not consider lower-level athletes (e.g., state and recreational levels). However, only elite athletes were involved in the “bubble” camp (i.e., to prepare for major events), and this opportunity facilitated a survey of a wide range of sports (including parasports), across sexes, by individuals rated as elite and word-class (~80% Olympic/Paralympic and/or world championship representatives). Moreover, the sample represented a majority of the country’s elite athletic population, and could be generalised to other (local) elite cohorts who travel often (e.g., training and competitions) and/or are exposed to lockdown-like and “bubble” situations. Additionally, we utilised changes in dietary habits to predict changes in perceived stress, which should be interpreted with caution, as changes in dietary habits could also occur as a result of changes in perceived stress (i.e., the opposite cause and effect relationship).

## 5. Conclusions

Increased perceived stress during lockdown was primarily mediated by the underlying sport classification, accounting for 16% of the increase (i.e., higher stress among para-athletes). Collectively, socio-demographic factors and improvements in training routines, well-being, and dietary habits explained 28% of decreased perceived stress during “bubble” camps. Dietary habits were negatively affected during lockdown, but this consequence was reversed (better) during the “bubble” camps. This study has provided useful insights into factors that increase perceived stress, and other related changes (e.g., diet), during germane situations. This study will help policy makers, sports organisations, and clubs to understand the effects of lockdowns on individual elite athletes, and therefore, to prepare appropriate measures. We recommend that interventions related to stress management, motivation, and athlete nutrition be tailored to sports/individual needs. Organisations should consider the use of “bubble” camps (i.e., potentially split into short “bubble” periods) during pandemics or lockdown-like situations.

## Figures and Tables

**Table 1:** Frequency analysis and perceived stress scores for different socio-demographic factors before and during a COVID-19 lockdown and a subsequent “Bubble” training camp in elite athletes. (mean ± SD).

Characteristic	Group			Perceived Stress Scores			(i) Δ, % *d*	(ii) Δ, % *d*	(iii) Δ, % *d*
		n	%	Prelockdown	Lockdown	“Bubble” Camp			
Sex	Male	53	70	17.8 ± 4.1	18.8 ± 4.3	17.0 ± 5.0	5.6 ^S^	−4.5 ^T^	−9.6 ^S^
	Female	23	30	17.8 ± 4.7	18.2 ± 5.3	17.7 ± 4.6	2.2 ^T^	−0.6 ^T^	−2.7 ^T^
				*p* = 0.979	*p* = 0.619	*p* = 0.604			
Sport classification	Able-bodied	47	62	17.7 ± 3.8	17.7 ± 4.1	17.0 ± 3.8	0.0 ^T^	−4.0 ^T^	−4.0 ^T^
	Parasports	29	38	17.9 ± 5.0	20.0 ± 5.1	17.5 ± 6.2	11.2 ^S^	−2.2 ^T^	−12.1 ^S^
				*p* = 0.874	***p* = 0.034**	*p* = 0.713			
Age	≤22 yr. old	22	29	17.2 ± 2.9	17.4 ± 3.3	16.4 ± 2.9	1.2 ^T^	−4.7 ^S^	−5.7 ^S^
	23–29 yr. old	41	54	18.7 ± 5.0	19.6 ± 5.2	18.2 ± 5.7	4.8 ^S^	−2.7 ^T^	−7.1 ^S^
	≥30 yr. old	13	17	16.1 ± 3.6	17.3 ± 3.9	15.4 ± 3.9	7.5 ^S^	−4.3 ^S^	−11.0 ^S^
				*p* = 0.144	*p* = 0.053	*p* = 0.176			
Experience	<10 years	41	54	18.6 ± 5.2	19.9 ± 5.1	17.5 ± 3.9	7.0 ^S^	−5.9 ^S^	−12.1 ^M^
	≥10 years	35	46	17.1 ± 3.3	17.5 ± 3.9	17.6 ± 6.1	2.3 ^T^	2.9 ^T^	0.6 ^T^
				*p* = 0.135	***p* = 0.025**	*p* = 0.552			
Marital status	Single	64	84	18.3 ± 4.4	18.9 ± 4.8	17.4 ± 5.0	3.3 ^T^	−4.9 ^T^	−7.9 ^S^
	Married	12	16	15.3 ± 2.6	16.9 ± 3.6	16.0 ± 4.2	10.5 ^M^	4.6 ^S^	−5.3 ^S^
				***p* = 0.029**	*p* = 0.178	*p* = 0.349			
Ethnicity/group	Malay	35	46	18.0 ± 4.3	18.8 ± 4.6	17.5 ± 5.1	4.4 ^T^	−2.8 ^T^	−6.9 ^S^
	Chinese	32	42	18.1 ± 4.2	18.4 ± 4.4	17.2 ± 4.5	1.7 ^T^	−5.0 ^S^	−6.5 ^S^
	EM Bumiputera	7	9	14.9 ± 4.3	17.4 ± 5.9	21.0 ± 5.7	16.8 ^S^	40.9 ^L^	20.7 ^M^
	Indian	2	3	20.0 ± 4.2	22.0 ± 7.1	14.4 ± 5.0	10.0 ^S^	−28.0 ^L^	−34.5 ^L^
				*p* = 0.259	*p* = 0.662	*p* = 0.301			

Note: (i) Δ prelockdown vs. lockdown, (ii) Δ prelockdown and “bubble” camp, (iii) Δ lockdown and “bubble” camp; magnitude of effect, *trivial* (^T^), *small* (^S^), *moderate* (^M^), *large* (^L^) change. EM Bumiputera: local ethnic groups of East Malaysia. Bolded *p*-Value indicates significant at *p* < 0.05.

**Table 2:** Summary of sequential regression predicting ∆ perceived stress for pre-to-during lockdown from socio-demographic, ∆ training routines and wellbeing, and ∆ dietary habit variables.

Model	Variables	UC		SC	T	*p*-Value	R	R^2^	Adj. R^2^	SEE	F	*p*-Value
		B	SE	β								
	(Constant)	−0.321	2.018		−0.159	0.874						
	Sport classification (able: 1, para: 2)	3.226	0.927	0.601	3.481	**0.001**	0.397					
1	Sex (female: 1, male: 2)	−0.421	0.682	−0.074	−0.617	0.539	0.097	0.19	0.14	2.45	3.45	0.009
	Age	−0.075	0.065	−0.149	−1.148	0.255	0.102					
	Experience (10y: 1, ≥10y: 2)	1.100	0.827	0.209	1.330	0.188	−0.169					
	Marital status (single: 1, married: 2)	0.648	0.847	0.090	0.765	0.447	0.133					
	(Constant)	−0.165	2.143		−0.077	0.939						
	Sport classification (able: 1, para: 2)	3.240	1.048	0.604	3.092	**0.003**	0.397					
	Sex (female: 1, male: 2)	−0.203	0.719	−0.036	−0.282	0.779	0.097					
	Age	−0.092	0.074	−0.183	−1.248	0.217	0.102					
	Experience (10y: 1, ≥10y: 2)	1.213	0.916	0.230	1.324	0.190	−0.169					
	Marital status (single: 1, married: 2)	0.393	0.915	0.055	0.429	0.669	0.133					
2	∆ Access to gym facilities	0.322	0.569	0.134	0.567	0.573	0.138	0.27	0.12	2.48	1.76	0.070
	∆ Access to sport-specific facilities	−0.576	0.491	−0.271	−1.172	0.246	0.066					
	∆ Access to recovery facilities	0.506	0.431	0.229	1.174	0.245	0.139					
	∆ Mental health	−0.372	0.709	−0.128	−0.525	0.602	0.102					
	∆ Emotional health	−0.476	0.563	−0.145	−0.846	0.401	0.035					
	∆ Training motivation	0.795	0.512	0.313	1.552	0.126	0.230					
	∆ Overall sleep quality	0.544	0.765	0.153	0.711	0.480	0.077					
	∆ Overall sleep quantity	−0.962	0.822	−0.257	−1.170	0.247	0.053					
	(Constant)	−0.835	2.327		−0.359	0.721						
	Sport classification (able: 1, para: 2)	3.380	1.142	0.630	2.960	**0.005**	0.397					
	Sex (female: 1, male: 2)	−0.068	0.752	−0.012	−0.090	0.929	0.097					
	Age	−0.078	0.081	−0.154	−0.959	0.342	0.102					
	Experience (10y: 1, ≥10y: 2)	1.181	0.993	0.224	1.189	0.239	−0.169					
	Marital status (single: 1, married: 2)	0.341	0.958	0.047	0.356	0.723	0.133					
	∆ Access to gym facilities	0.508	0.616	0.212	0.825	0.413	0.138					
	∆ Access to sport-specific facilities	−0.608	0.530	−0.286	−1.147	0.256	0.066					
	∆ Access to recovery facilities	0.501	0.461	0.227	1.089	0.281	0.139					
3	∆ Mental health	−0.663	0.856	−0.228	−0.774	0.442	0.102	0.30	0.063	2.56	1.27	0.242
	∆ Emotional health	−0.291	0.692	−0.089	−0.421	0.675	0.035					
	∆ Training motivation	0.793	0.550	0.312	1.442	0.155	0.230					
	∆ Overall sleep quality	0.565	0.870	0.159	0.649	0.519	0.077					
	∆ Overall sleep quantity	−0.879	0.941	−0.235	−0.933	0.355	0.053					
	∆ Body weight	−0.010	0.118	−0.010	−0.084	0.933	−0.121					
	∆ Daily amount of drink/water	0.498	0.833	0.097	0.598	0.552	−0.098					
	∆ Numbers of daily meal	−0.218	0.569	−0.060	−0.384	0.702	−0.051					
	∆ Dinner within 2 h of bedtime	−0.031	0.604	−0.008	−0.052	0.959	0.034					
	∆ Late night snack	0.091	0.614	0.022	0.149	0.882	0.088					
	∆ Skip breakfast	0.643	0.541	0.156	1.188	0.240	0.102					
	(Constant)	−0.065	0.360		−0.181	0.857						
4	Sport classification (able: 1, para: 2)	2.132	0.573	0.397	3.723	**0.001**	0.397	0.16	0.15	2.44	13.9	0.001

Note: UC: unstandardised coefficients; SC: standardised coefficients; SE: standard error; R: coefficient of correlation; R^2^: coefficient of determination; adj. R^2^: adjusted R^2^; SEE: standard error of the estimate. Able: able-bodied; para: parasports. Bolded *p*-Value indicates significant at *p* < 0.05.

**Table 3:** Summary of sequential regression predicting ∆ perceived stress for during lockdown-to-“bubble” camp from socio-demographic, ∆ training routines and wellbeing, and ∆ dietary habit variables.

Model	Variables	UC		SC	T	*p*-Value	R	R^2^	Adj. R^2^	SEE	F	*p*-Value
		B	SE	β								
	(Constant)	−1.754	3.098		−0.566	0.573						
	Sport classification (able: 1, para: 2)	−1.379	1.423	−0.177	−0.969	0.336	−0.252					
1	Sex (female: 1, male: 2)	−0.818	1.049	−0.100	−0.780	0.438	−0.152	0.09	0.02	3.77	1.31	0.270
	Age	−0.004	0.100	−0.006	−0.041	0.968	−0.040					
	Experience (10y: 1, ≥10y: 2)	0.668	1.271	0.088	0.525	0.601	0.233					
	Marital status (single: 1, married: 2)	1.170	1.306	0.113	0.895	0.374	0.054					
	(Constant)	−1.551	3.255		−0.477	0.635						
	Sport classification (able: 1, para: 2)	−1.853	1.639	−0.239	−1.130	0.263	−0.252					
	Sex (female: 1, male: 2)	−0.273	1.152	−0.033	−0.237	0.814	−0.152					
	Age	−0.010	0.110	−0.014	−0.092	0.927	−0.040					
	Experience (10y: 1, ≥10y: 2)	−0.207	1.397	−0.027	−0.148	0.883	0.233					
	Marital status (single: 1, married: 2)	1.650	1.358	0.160	1.215	0.229	0.054					
2	∆ Access to gym facilities	−0.163	0.622	−0.061	−0.262	0.794	0.099	0.25	0.09	3.64	1.55	0.125
	∆ Access to sport-specific facilities	−0.996	0.591	−0.373	−1.687	0.097	−0.011					
	∆ Access to recovery facilities	0.258	0.486	0.093	0.531	0.598	0.181					
	∆ Mental health	0.934	0.797	0.238	1.172	0.246	0.304					
	∆ Emotional health	0.232	0.713	0.057	0.325	0.746	0.312					
	∆ Training motivation	0.662	0.746	0.173	0.888	0.378	0.184					
	∆ Overall sleep quality	−0.039	0.958	−0.010	−0.041	0.968	0.211					
	∆ Overall sleep quantity	0.759	1.333	0.157	0.570	0.571	0.291					
	(Constant)	−2.545	3.638		−0.700	0.487						
	Sport classification (able: 1, para: 2)	−2.191	1.779	−0.282	−1.231	0.223	−0.252					
	Sex (female: 1, male: 2)	0.085	1.282	0.010	0.066	0.947	−0.152					
	Age	−0.015	0.117	−0.021	−0.127	0.899	−0.040					
	Experience (10y: 1, ≥10y: 2)	0.200	1.617	0.026	0.123	0.902	0.233					
	Marital status (single: 1, married: 2)	1.546	1.503	0.150	1.028	0.308	0.054					
	∆ Access to gym facilities	−0.376	0.666	−0.141	−0.564	0.575	0.099					
	∆ Access to sport-specific facilities	−0.842	0.666	−0.316	−1.264	0.212	−0.011					
	∆ Access to recovery facilities	0.192	0.527	0.069	0.365	0.717	0.181					
3	∆ Mental health	0.993	0.829	0.253	1.198	0.236	0.304	0.28	0.21	3.75	1.115	0.363
	∆ Emotional health	0.582	0.826	0.143	0.705	0.484	0.312					
	∆ Training motivation	0.558	0.816	0.146	0.684	0.497	0.184					
	∆ Overall sleep quality	−0.300	1.073	−0.078	−0.280	0.781	0.211					
	∆ Overall sleep quantity	1.011	1.497	0.209	0.675	0.502	0.291					
	∆ Body weight	−0.176	0.268	−0.090	−0.657	0.514	−0.085					
	∆ Daily amount of drink/water	0.774	0.889	0.126	0.871	0.388	−0.087					
	∆ Numbers of daily meal	−0.427	0.679	−0.098	−0.629	0.532	0.019					
	∆ Dinner within 2 h of bedtime	−0.140	0.662	−0.037	−0.211	0.834	−0.003					
	∆ Late night snack	−0.128	0.619	−0.036	−0.206	0.838	0.000					
	∆ Skip breakfast	0.138	0.484	0.041	0.286	0.776	0.094					
	(Constant)	−0.784	0.571		−1.374	0.174						
	Sport classification (able: 1, para: 2)	−1.691	0.863	−0.218	−1.959	0.054	−0.216					
4	∆ Access to sport-specific facilities	−0.596	0.329	−0.223	−1.811	0.074	−0.204	0.17	0.13	3.55	4.798	0.004
	∆ Mental health	1.436	0.487	0.365	2.949	**0.004**	−0.332					

Note: UC: unstandardised coefficients; SC: standardised coefficients; SE: standard error; R: coefficient of correlation; R^2^: coefficient of determination; adj. R^2^: adjusted R^2^; SEE: standard error of the estimate. Able: able-bodied; para: parasports. Bolded *p*-Value indicates significant at *p* < 0.05.

## Data Availability

The data supporting the conclusions of this article will be made available by the corresponding author, upon reasonable request.
